# Research Knowledge and Skills Among Medical and Allied Health Students Attending a Summer Research Course: A Pretest and Posttest Analysis

**DOI:** 10.7759/cureus.3132

**Published:** 2018-08-11

**Authors:** Mohamad Al-Tannir, Amani Abu-Shaheen, Saleh AlSumaih, Nawaf F AlMukaibil, Ryan AlHarbi, Humariya Heena, Luai Sallout, Adeebah Mahha, Nasser Mohammed Marran, Isamme AlFayyad

**Affiliations:** 1 Research Center, King Fahad Medical City, Riyadh, SAU; 2 College of Medicine, King Faisal University, Al-Ahsa, SAU; 3 College of Medicine, Al-Imam Mohammad Ibn Saud Islamic University, Riyadh, SAU; 4 College of Medicine, King Faisal University, Riyadh, SAU; 5 College of Medicine, Alfaisal University, Riyadh, SAU; 6 College of Medicine, Jazan University, Riyadh, SAU; 7 College of Medicine, Jazan University, Sabya, SAU

**Keywords:** knowledge, medical students, research course, skills

## Abstract

Introduction

This study assessed the impact of a summer research training course on the knowledge levels and skills of medical and allied health students.

Methods

A one group pretest-posttest quasi-experiment study was conducted during a summer research course at King Fahad Medical City, Saudi Arabia. A self-administered questionnaire was distributed among course participants twice, on the first day (pretest) and the last day of the course (posttest). The questionnaire consisted of four sections: study design, literature review, formulation of a research question, and biostatistics.

Results

A total of 44 participants were included in the study, of whom 27 (61.4%) were medical students. The overall mean score of correct responses of the participants was 17.70±4.00 in the pretest compared to 22.18±6.64 in the posttest (p<0.001). The mean score of the correct responses of the participants in the “study design” pretest section was (4.23±1.51) compared to (10.23 ± 3.71) in the posttest, (p<0.001). While for the literature review section, the mean score of the correct responses in the pretest was (2.20±1.19) and (2.77±1.34) in the posttest, (p=0.027). Moreover, our results revealed that all participants (100%) were able to execute all the steps of a research project and 6 (13.64%) participants were able to submit papers for publication.

Conclusions

Our results showed that a research training program might enhance research knowledge and skills in terms of the successful accomplishment of relevant assessment tasks among medical and allied health students.

## Introduction

Given the growing interest in and recognition of research training programs, research training courses become an opportunity to provide medical and allied health students with extensive and practical research experience. A research training course is an essential item of medical education and a crucial opportunity to develop future physicians and healthcare providers with excellent research skills [1-2]. Previous studies reported that research experience throughout college years is strongly associated with more postgraduate researchers and future research achievements [1-3]. Additionally, conducting a research project helps students to develop critical thinking and the ability to evaluate literature efficiently. It also supports the establishment of problem-solving, teamwork, and leadership skills [4-5]. However, the conduct of a scientific research project entails an understanding of various phases of the research project; starting from formulating a research question through conducting an efficient literature review and appraisal, developing the appropriate study methodology, as well as accomplishing data collection and analysis within a specified time frame, and finally acquiring and practicing the skills of manuscript writing [3].

Preparing students from different healthcare specialties by inculcating adequate information and research skills fosters a positive attitude towards research among students at the early stages of their career [6]. Furthermore, with the increasing emphasis on the enhancement of the research culture, the promotion of training in healthcare research is necessary to combat the growing need for researchers in all medical fields. Consequently, there is a need for effective research courses and programs at undergraduate levels, which can be vital in making a workforce of experienced researchers in the Kingdom of Saudi Arabia. However, despite the presence of some undergraduate research educational programs, limited published reports illustrate, in detail, the development and execution of the programs and less have research evidence of their educational impact [7-8]. Therefore, this study aimed to assess the impact of summer research training courses on the knowledge levels and skills of the medical and allied health students.

## Materials and methods

Study design and setting

After receiving institutional review board approval, a one-group pretest-posttest quasi-experiment study was conducted during a summer research course at Research Center, King Fahad Medical City, Riyadh, Saudi Arabia.

Participants

All participants of the summer research course who agreed voluntarily were eligible to participate in this study. Participants who either skipped the first day of the course or did not attend the posttest session were excluded.

Intervention

The educational interventions were the summer research course in the form of research lectures and hands-on workshops. The content of the courses was based on a review of the literature, covering the basic topics in the research field [9-10]. The courses’ curriculum covered study design, literature review, the formulation of a research question, and basic biostatistics and had 20 sessions: two hours/session for four weeks.

Data collection

For knowledge measurement, a self-administered assessment questionnaire was distributed twice on the first day (pretest) and the last day of the course (posttest) with a four weeks interval. The assessment questionnaire was formulated according to Downing’s guidelines for effective test development [11-12] and consisted of 45 multiple-choice questions about the essential components of a research pathway, which included four sections: study design, literature review, formulation of a research question, and biostatistics. The study design section included questions for identifying the appropriate study design (covering observational and experimental studies) and other questions related to blinding, randomization, bias and bias elimination, sampling techniques, confounding factors, and survey development reliability testing. In the literature review section, the students were asked about the purposes and steps of conducting a literature review and the best evidence of resource utilization in the literature review. The third section included questions about variables and criteria for formulating a research question. While the biostatistics section included questions related to Statistical Package for the Social Sciences (SPSS) (IBM Corp., Armonk, NY, US), descriptive and inferential statistics, correlational tests, and hypothesis testing. Moreover, demographic data were collected. For calculating the participants’ knowledge score, correct answers were given a score of one while incorrect answers or unanswered questions were given a score of zero. The total score of the correct responses reflected the students’ knowledge level. The assessment questionnaire had been used previously in our courses, with high reliability; Cronbach’s alpha is 0.94.

For skills assessment, the participants were asked to come up with a research question, writing a full research proposal, submitting it to the institutional review board, collecting data, and presenting their studies.

Ethical considerations

The study protocol was evaluated and approved by the IRB at King Fahad Medical City, Riyadh, Saudi Arabia. Written informed consent was obtained from each participant before enrollment in the study.

Statistical analysis

Data were analyzed using the SPSS version 22.0 (IBM Corporation, Armonk, NY, US). Categorical variables, such as gender, specialty, and year of education, were presented in frequencies and percentages. Whereas the continuous variable of pretest and posttest scores of the summer course were expressed as mean ± SD, the independent sample t-test/analysis of variance (ANOVA) was used to determine the mean significant differences in pretest and posttest scores. The paired sample t-test/Wilcoxon signed ranked test was applied to determine the mean and median significant differences between pretest and posttest scores. P-value < 0.05 was considered statistically significant.

## Results

The total number of participants in the research summer course was 50; of whom, 44 students completed both the pretest and the posttest, which indicated a response rate of 88%. The majority of the participants 27 (61.4%) were medical students and 25 (56.8%) were female.

Research knowledge levels among medical and allied health students

Our results showed that the participants' pretest mean score of correct responses in “study design” was (4.23±1.51) while the posttest mean score was 10.23±3.71. The pretest mean score for correct responses for all participants in the “literature review” section was 2.20±1.19 and the posttest mean was 2.77±1.34. Regarding the scores of the participants in the “biostatistics” section, the mean score was 4.23±1.51 in the pretest while the posttest mean score was 4.43±1.55. Moreover, in the “research question” section, the pretest mean score was 4.32±1.52 and the posttest mean score was 4.75±1.74.

Our results showed that there was a statistically significant difference in the pretest and posttest mean scores in the study design and literature review sections (p<0.001) and (p=0.027); respectively (Table 1).

**Table 1:** Pretest and posttest mean score of knowledge levels for each course's section

	Median ( Minimum-Maximum)	Mean ± S.D	P-value
Pretest study design score	4 (2-7)	4.23±1.51	< 0.001
Posttest study design score	10.50 (2-16)	10.23±3.71	
Pretest literature review score	2 (0-5)	2.20±1.19	0.027
Posttest literature review score	3 (1-6)	2.77±1.34	
Pretest research question	4 (2-9)	4.32±1.52	0.156
Posttest research question	4.50 (1-8)	4.75±1.74	
Pretest biostatistics score	4 (2-7)	4.23±1.51	0.476
Posttest biostatistics score	5 (1-7)	4.43±1.55	

After calculating the knowledge score for all sections before and after the course, our results showed that the overall mean score of the correct responses of the study participants was 17.70±4.00 in the pretest as compared to 22.18±6.64 in the posttest (p<0.001) (Table 2).

**Table 2:** Overall pretest and posttest mean knowledge scores of summer research course students

	Median (Minimum-Maximum)	Mean ± S.D	P-value
Pre Total Test Score	18 (10 -26)	17.70±4.00	<0.001
Post Total Test Score	23 (8 -33)	22.18±6.64	

Research skills among medical and allied health students 

Our results revealed that all students 44 (100%) were able to execute all the steps of a research project, from conception to successful institutional review board submission of the proposal, and to present their studies to professional audiences. Moreover, six (13.64%) students were able to submit their manuscript for publication.

## Discussion

In line with the Kingdom of Saudi Arabia's national vision 2030, it is essential to allocate resources to basic and clinical research to fulfill the changing needs of the population regarding healthcare due to its unique demography, culture, and traditions. Our results showed that a statistically significant difference was noticed in the knowledge after conducting the research training course. The training program showed a marked increase in the participants' knowledge score from 17.70±4.00 to 22.18±6.64. Similarly, a study conducted at King Abdulaziz University revealed that participants’ mean knowledge score was improved significantly after the conduct of a research undergraduate program [3]. Furthermore, research workshops have been shown to improve the medical students' research knowledge considerably [1-3,7,9].

The key indicator of success was that the students were able to execute all the steps of a research project, from conception to successful institutional review board submission of the proposal, thereby narrowing the gap between theory and practice. Another indicator reflecting the improvement of the participants’ knowledge and skills was their success in presenting their research to professional audiences with different study designs, including case reports and cross-sectional and experimental studies. Moreover, six (13.64%) students were able to submit their manuscript for publication. Similarly, other studies reported that 5% to 25% of program completers were able to publish their research while they are still in medical school [11-12].

The lack of research exposure and training during student years in the medical field accentuates the need to modify undergraduate curricula so that some short-term mandatory research educational intervention is incorporated. Learning opportunities should be augmented to increase research activity in the country, both in terms of quality and quantity. Further improvement in students' research activities could be through frequent conducting research workshops in medical schools and reserving time for such activities by integrating them into the curriculum of the training program.

Students' research projects should be encouraged, and their participation in mentorship programs should be boosted. A healthy research environment through the conduct of short summer research courses can encourage them to pursue a successful research career.

The key purpose of conducting the pretest-posttest questionnaire in the summer course is to find areas of improvement. The students were expected, through their participation in the summer course, to gain research knowledge and skills in the conduct of clinical research, data collection, writing of scientific articles or reviews in their own research interest, the ability to recapitulate and discuss research results, and, ultimately, present their researches in an official and recognized oral or poster presentation to professional researchers at the study site. These expectations were soundly aligning with the pre-settled course objectives. Also, the findings of this study aim to stimulate the conduct of more research training programs throughout the kingdom. This study can also serve as an inspiring step towards the integration of research with medical education.

The limitations of this study were that it was unicentered and the study sample was relatively small. Moreover, our study did not include a control group and had a short-term outcome, so it is hard to know the true impact of this research education intervention. Thus, more research on the long-term effectiveness of research training is needed, incorporating a cohort group and a larger study sample.

## Conclusions

Our results showed that a research training program might enhance research knowledge and skills in mainly study design and literature review in terms of the successful accomplishment of relevant assessment tasks among medical and allied health students. Based on the study findings, we strongly endorse that a similarly structured research summer course be implemented annually, to provide additional opportunities for students to advance their exposure to research at the appropriate stage of their academic endeavors.

## References

[1] Khan H, Taqui AM, Khawaja MR, Fatmi Z (2007). Problem-based versus conventional curricula: Influence on knowledge and attitudes of medical students towards health research. PLoS One.

[2] Reinders JJ, Kropmans TJB, Cohen-Schotanus J (2005). Extracurricular research experience of medical students and their scientific output after graduation. Med Educ.

[3] Becher Al-Halabi YM, Hasan M, Alkhadhari S ( 2014). Extracurricular research activities among senior medical students in Kuwait: experiences, attitudes, and barriers. Adv Med Educ Pract.

[4] Griffin MF, Hindocha S (2011). Practices of medical students at British medical schools: experience, attitudes and barriers to publish. Med Teach.

[5] Ibrahim N, Fetyani D, Bashwari J (2013). Assessment of the research-oriented knowledge, attitude and practice of medical students and interns of the King Abdulaziz University, Jeddah and the adoption of a research-intervention educational program. Rawal Med J.

[6] Devi V, Abraham RR, Adiga A, Ramnarayan K, Kamath A (2012). Fostering research skills in undergraduate medical students through Mentored Student Projects: example from an Indian medical school. Kathmandu Univ Med J.

[7] Mullan JR, Weston KM, Rich WC, McLennan PL (2014). Investigating the impact of a research-based integrated curriculum on self-perceived research experiences of medical students in community placements: a pre-and post-test analysis of three student cohorts. BMC Med Educ.

[8] Black ML, Curran MC, Golshan S, Daly R, Depp C, Kelly C, Jeste DV (2013). Summer research training for medical students: impact on research self‐efficacy. Clin Transl Sci.

[9] Ramjiawan B, Pierce G, Anindo M (2012). An international basic science and clinical research summer program for medical students. Adv Physiol Educ.

[10] Downing M (2006). Twelve steps for effective test development. Handbook of Test Development.

[11] Kalyuga S, Chandler P, Sweller J (1998). Levels of expertise and instructional design. Hum Factors.

[12] Gonzales A, Westfall J, Barley G (1998). Promoting medical student involvement in primary care research. Fam Med.

